# Fixation of Crescent Pelvic Fracture in a Tertiary Care Hospital: A Steep Learning Curve

**DOI:** 10.7759/cureus.5614

**Published:** 2019-09-10

**Authors:** Asif Jatoi, Badaruddin Sahito, Dileep Kumar, Nauman H Rajput, Maratib Ali

**Affiliations:** 1 Orthopaedics, Dr. Ruth K.M. Pfau Civil Hospital, Karachi, PAK; 2 Orthopaedics, Dow University of Health Sciences, Karachi, PAK; 3 Orthopaedic Surgery, Dr. Ruth K.M. Pfau Civil Hospital, Dow University of Health Sciences, Karachi, PAK

**Keywords:** crescent fracture, instability, fixation, percutenous screws, plates

## Abstract

Background

Crescent fracture-dislocation of sacroiliac joint is a type of lateral compression pelvic injury associated with instability. These fractures comprise 12% of lateral compression fractures.

Objective

The objective of this study is to share the experience and to assess the functional outcome of fixation in crescent fracture-dislocation.

Methods

We analyzed a descriptive case series with clinical data of 15 patients at the Department of Orthopedics Surgery at the Dr. Ruth K.M. Pfau Civil Hospital at Dow University of Health Sciences in Karachi, Pakistan, from January 2016 to August 2018. The patients were treated by closed and open fracture reduction and fixed with percutaneous screws and reconstruction plates.

Results

A total of 15 patients were included in this study with age ranging from 20 to 60 years (11 men [73%]; four women [27%]). According to the mechanism of injury, five (33%) had motorcycle accidents; four (27%) had collision while sitting in a car; three (20%) were pedestrians hit by a vehicle; four (27%) were injured while sitting in van; two (13%) had bus-related injury, and one (6.5%) presented with a history of wall collapse.

Five (33%) patients had type I fractures, seven (47%) had type II fractures, and three (20%) had type III fractures Associated injuries were midshaft femur fracture in two patients, contralateral superior and inferior rami fracture in three patients, and open tibia fracture in one patient. All fractures were fixed with reconstruction plates and screws. Patients were kept as non-weight-bearing on the injured joint for three weeks, mobilized non-weight-bearing on the contralateral leg after three weeks, and partial weight-bearing was started at eight weeks; full weight-bearing was started after three months. Nine patients (60%) had excellent outcomes, three (20%) had a good outcome, and three (20%) had a poor outcome.

Conclusion

Crescent fracture-dislocations are unstable injuries. These fractures should have proper reduction and fixation that will reduce pain, malunion, and shortening.

## Introduction

The pelvis is a ring-like structure that bridges the axial skeleton and the lower extremities. Following high-velocity trauma, many patients usually present with pelvic fractures. Pelvic fractures are rare injuries which occur at a frequency of approximately 20 to 37 per 100,000 people. The frequency as a percentage of all fractures lies between 0.3% and 6%. In polytrauma, pelvic ring injury is present in 20% of cases [1]. Crescent fracture-dislocation is a type of pelvic fracture that occurs as a result of lateral compression force. Crescent fracture-dislocation is characterized by variable disruption of the inferior part of the sacroiliac joint, while the superior and posterior joint line remains intact [2]. The posterior superior iliac spine remains firmly attached to the sacrum via a superior portion of the posterior ligamentous complex; this makes the hemipelvis rotationally unstable [3,4]. However, because of the muscular pelvic floor and the sacrospinous and sacrotuberous ligamentous remain intact, the hemipelvis is stable to vertical forces [5-7].

Half of pelvic injuries are lateral compression type, and 12% accounts for crescent fracture-dislocation [6-8]. The Day et al. classification system groups crescent fractures into three distinct types [6]. According to Khaled, type I fractures involve only one-third of the sacroiliac joint. Type II fractures involve between one and two-thirds of the sacroiliac joint. Type III involves more than two-third of the sacroiliac joint [9].

These fractures are unstable injuries, so it is essential to maintain the sacroiliac joint congruency with fixation and to prevent pain, malunion, sacroiliac joint arthritis, and seating obliquity [5,10]. The anterior or posterior approach can be used according to the type of fracture [11]. Percutaneous iliosacral screw can be used for type I, and reconstruction plate with or without iliosacral screws can be used to fix type II and III crescent fracture-dislocations [9,12,13].

## Materials and methods

We conducted a review of clinical data of 15 patients at the Department of Orthopedics Surgery at the Dr. Ruth K.M. Pfau Civil Hospital at Dow University of Health Sciences in Karachi, Pakistan, from January 2016 to August 2018. The patients were treated by closed and open reduction of the fracture and fixed with percutaneous screws and reconstruction plates. All patients aged 20 to 60 years admitted through accident and emergency with crescent fracture-dislocation were included in the study. Patients were excluded if they had associated acetabular fractures, open book pelvic fractures, associated hip dislocation, or isolated pubic rami fractures. 

## Results

**Figure 1:**
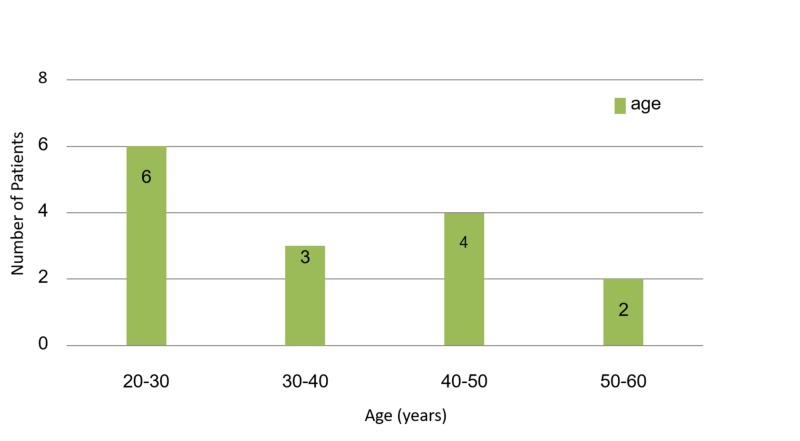
Age distribution

A total of 15 patients were included in this study: 11 were men (73%), and four (27%) were women. Figure 1 presents age distribution of the study population.

**Figure 2:**
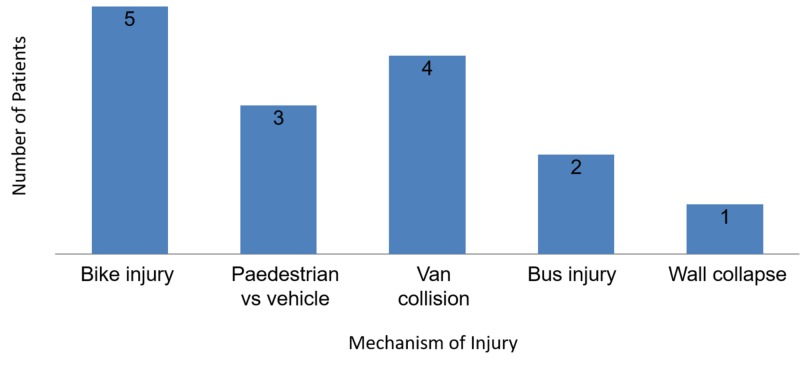
Mechanism of injury

 Figure 2 presents the distribution of injuries according to mechanism of injury.

Fracture configuration was evaluated with a full pelvic x-ray with anteroposterior view and inlet and outlet views along with CT scan with three-dimensional reconstruction. Table 1 presents fracture types according to the Day classification [6].

**Table 1:** Fracture type according to the Day et al. classification

Day Classification [6]	N	Percentage
Type 1	5	33%
Type 2	7	47%
Type 3	3	20%

Patients were kept non-weight-bearing for three weeks, mobilized non-weight-bearing the contralateral leg on the following three weeks, and while partial weight-bearing was started eight weeks postoperatively, full weight-bearing was started three months postoperatively. Outcomes were assessed using the Majeed Outcome Scale (Table 2) [14].

**Table 2:** Outcome assessment according to Majeed Outcome Scale

Pain (30 Points) [14]	Score	Standing (36 Points) [14]	Score
Intense, continuous at rest	0-5	A walking aids	
Intense with activity	10	Bedridden or almost	0-2
Tolerable, but limits activity	15	Wheelchair	4
With moderate activity, abolished by rest	20	Two crutches	6
Mild, intermittent, normal activity	25	Two sticks	8
Slight, occasional, or no pain	30	One stick	10
		No sticks	12
Work (20 points) [14]		B gait unaided	
No regular work	0-4	Cannot walk or almost	0-2
Light work	8	Shuffling small steps	4
Change of job	12	Gross limp	6
Same job, reduced performance	16	Moderate limp	8
Same job, same performance	20	Slight limp	10
Sitting (10 points) [14]		Normal	12
Painful	0-4		
Painful if prolonged or awkward	6	C walking distance	
Uncomfortable	8	Bedridden or few meters	
Free	10	Very limited time and distance	0-2
Sexual intercourse (4 points) [14]		Limited with sticks, difficult without	4
Painful	0-I	0-1 Prolonged standing possible	6
Painful if prolonged or awkward	2	2 One hour with a stick limited without	8
Uncomfortable	3	3 One hour without sticks slight pain or limp	10
Free	4	4 Normal for age and general condition	12

The patients with a score more than 85 were graded as excellent, patients with a score between 55 and 84 were graded as good, and patients with a score less than 54 were graded as poor outcome.

The operative records, postoperative images, and follow-up records, as well as their final outcomes, were recorded. Of 15 patients assessed, nine (60%) had an excellent outcome, three (20%) had a good outcome, and three (20%) had a poor outcome. Out of 15 patients, one had L5 nerve root injury, while one had postoperative wound complication.

## Discussion

Crescent fracture-dislocation needs obligatory anatomic reduction to maintain the joint congruity to prevent posttraumatic arthritis. Holdsworth et al. reported that greater than 50% of patients with crescent fracture treated conservatively were unable to perform heavy manual work and had persistent posttraumatic arthritic pain [11]. Park et al. treated these injuries surgically to mobilize patients early, using plates and screws for fixation [12]. With anterior plating on the sacroiliac joint, the L5 nerve root needs to be respected [12]. In our study, partial weight -bearing started at six weeks, while full weight-bearing started at 12 weeks. Out of 15 patients, one had L5 nerve root injury.

Borrelli et al. used a simple posterolateral approach to provide direct visualization and anatomic reduction of the sacroiliac joint [3]. With subsequent stabilization using extra-articular lag screws and neutralization, plates achieve union rate with a low incidence of complications and acceptably low morbidity [3]. We used anterior approach for type I and II fractures, while posterolateral approach for type III fractures. One patient with posterior approach had postoperative wound complication.

Khaled et al. treated 66 patients with lateral compression pelvic fractures with percutaneous iliosacral screws in 20 fractures, plates in 40 fractures, combined iliosacral screws with plates in four fractures, and they added lateral compression screws in two of the cases. Most type I fractures were fixed by open reduction internal fixation (ORIF) using plate and screws, while most of the type II and III fractures were stabilized with iliosacral screws fixation percutaneously. The clinical results were good in all cases and pain-free with movement. Radiographic union was noted in 100% of Khaled’s cases [9]. Similarly, we used anterior plate fixation for type I, while type II and III fractures stabilized with posterior plate fixation with percutaneous iliosacral screws.

Chen et al. treated 58 patients with unstable posterior pelvic ring fracture. Internal fixation with percutaneous reconstruction plate via posterior approach was used in 29 patients (group A), and internal fixation with percutaneous sacroiliac screws via posterior approach was used in 29 patients (group B). This showed excellent and good rates of 86.1% for percutaneous reconstruction plate and 88.2% for percutaneous sacroiliac screws, with group B having little more risk of nerve and vessel injury [15].

Starr et al. treated 27 patients with crescent fracture-dislocation with closed reduction. All patients were followed up clinically until fracture union, except for two patients who were lost to early follow-up. Of 27, 16 (59%) returned to work. All the patients were ambulatory without assistive devices, except for a 89-year-old female patient who uses a walker. One patient experienced occasional hardware pain associated with weather changes and underwent hardware removal [16]. All patients in our study achieved fracture union.

Shui et al. conducted a retrospective study of 117 fracture patients and found that functional recovery was better in those who underwent closed reduction and percutaneous screw fixation than those who underwent ORIF (P<0.01) [8].

Another study by Khaled included 43 patients who had sustained lateral compression (crescent) pelvic fractures (44 fractures). Percutaneous iliosacral screws alone were used in 20 fractures, plates alone were used in 22 fractures, and both plates and iliosacral screws were used in two fractures. The operative time was shorter for cases treated with iliosacral screws (40 minutes; range: 30 to 60 minutes) than that for cases treated with plate fixation (100 minutes; range: 60 to 150 minutes). The difference was statistically significant (P<0.001). The clinical results were good in all cases, and there were no wound complications, neurological complications, or residual rotational deformity of the limb. The healing rate was 100% [13].

Yinger et al. reviewed mechanical testing of posterior pelvic ring fixation, and they selected commonly used fixation implants in nine different combinations. Their findings suggest that the addition of a single iliosacral screw increases the stiffness of other fixation modes of posterior ring. Two iliosacral screws and the combination of an iliosacral screw and anterior sacroiliac joint plate provide the best potential for fixation stiffness [17]. In contrast, we used single iliosacral screw as putting two screws was technically demanding.

Our study had several limitations. Our study population was small, with a short follow-up period. Also, our study was a single-institution study with one surgeon; thus, comparisons were made with control groups in previously published studies, making generalization of our findings to a wider population limited [6,9,15].

## Conclusions

Crescent fracture-dislocations are unstable injuries. The surgical approach is dependent on sacroiliac joint articular alignment. Besides the fracture configuration, additional factors like delay in surgery, locking of the fracture fragments, and comminution of the iliac or sacral fragment, as well as access to the additional injuries, contribute to clinical decision making. These fractures should have proper reduction and fixation that will reduce pain, malunion, and shortening.
